# Supplementary material for: A smart pathogen detector engineered from intracellular hydrogelation of DNA-decorated macrophages
Source: Nat Commun. 2023 May 22;14:2927. doi: 10.1038/s41467-023-38733-w (PMC10203291; doi:10.1038/s41467-023-38733-w)
Supplement: Supplementary file 1 — Supplementary Information [file 41467_2023_38733_MOESM1_ESM.pdf]

Supplementary Information

A Smart Pathogen Detector Engineered from Intracellular Hydrogelation of DNA-Decorated Macrophages

Yueyue Gui [1,#], Yujing Zeng [2,#], Binrui Chen [1], Yueping Yang [1], Jiehua Ma [3], Chao Li [1,*]

[1]School of Food and Biological Engineering, Hefei University of Technology, Hefei, 230009, P. R. China.
[2]State Key Laboratory of Analytical Chemistry for Life Science, School of Life Sciences, Nanjing University, Nanjing, 210023, P. R. China.
[3]Tongren Hospital, Shanghai Jiao Tong University School of Medicine, Shanghai, 200336, P. R. China.

[*]E-mail: lchao@hfut.edu.cn (C. Li)

[#]These authors contributed equally: Yueyue Gui, Yujing Zeng

Table of contents

Supplementary Table 1 .... S-1
Supplementary Fig. 1 .... S-2
Supplementary Fig. 2 .... S-3
Supplementary Fig. 3 .... S-4
Supplementary Fig. 4 .... S-5
Supplementary Fig. 5 .... S-6
Supplementary Fig. 6 .... S-7
Supplementary Fig. 7 .... S-8
Supplementary Fig. 8 .... S-9
Supplementary Fig. 9 .... S-10
Supplementary Fig. 10 .... S-11
Supplementary Fig. 11 .... S-12
Supplementary Fig. 12 .... S-13
Supplementary Fig. 13 .... S-14
Supplementary Fig. 14 .... S-15
Supplementary Fig. 15 .... S-16
Supplementary Fig. 16 .... S-17
Supplementary Fig. 17 .... S-18
Supplementary Fig. 18 .... S-19

Supplementary Table 2 ..... S-20
Supplementary Fig. 19 ..... S-21
Supplementary Fig. 20 ..... S-22
Supplementary Fig. 21 ..... S-23
Supplementary Fig. 22 ..... S-24
Supplementary Table 3 ..... S-25
Supplementary Fig. 23 ..... S-26
Supplementary Fig. 24 ..... S-27
Supplementary Fig. 25 ..... S-28
Supplementary Fig. 26 ..... S-29
Supplementary Fig. 27 ..... S-30
Supplementary Fig. 28 ..... S-31
Supplementary References S-32

Supplementary Table 1 The used oligonucleotides in this study.

Name	Sequence (5'→3')
Ch/FITC-DNA1	Cholesterol-GATGTGTGCGTTGTCGAGACCTGCGACCGG AA-FITC
Ch/FITC-DNA2	TTCCGGTCGCAGGTCTCGACAACGCACACATC-Cholesterol
FITC-DNA1	GATGTGTGCGTTGTCGAGACCTGCGACCGGAA-FITC
FITC-DNA2	TTCCGGTCGCAGGTCTCGACAACGCACACATC
E. coli DNAzyme	FITC-TGTCGAGACCTGCGACAGGAAGACTACACACAGTTGTGTG-Cholesterol
E. coli substrate	Cholesterol-ACTCTTCCTAGCTrATGGTTCGATCAAGA-BHQ1
Random DNA	Cholesterol-AGACCTGCGACATGTCGAGACCTCACAGTTGTGTA
S. aureus DNAzyme (Rh)	Cholesterol-CTATGAACTGAC/Rh/TrAT/BHQ2/GACCTCACTACCAAGA TGCCATCCTACCAACCACGAAGTACATTTCAAACTCATAACAATCCATCG GTTAGGTCCTGGTTGG-Cholesterol
S. aureus DNAzyme (FITC)	Cholesterol-CTATGAACTGAC/FITC/TrAT/BHQ1/GACCTCACTACCAAG ATGCCATCCTACCAACCACGAAGTACATTTCAAACTCATAACAATCCATC GGTTAGGTCCTGGTTGG-Cholesterol

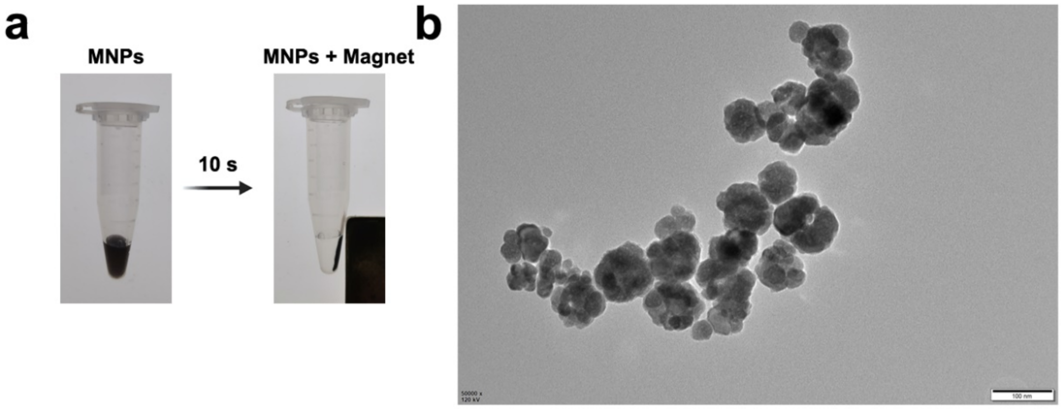

Supplementary Fig. 1: (**a**) Photograph of magnetic nanoparticles (MNPs) solution and (**b**) its TEM image.

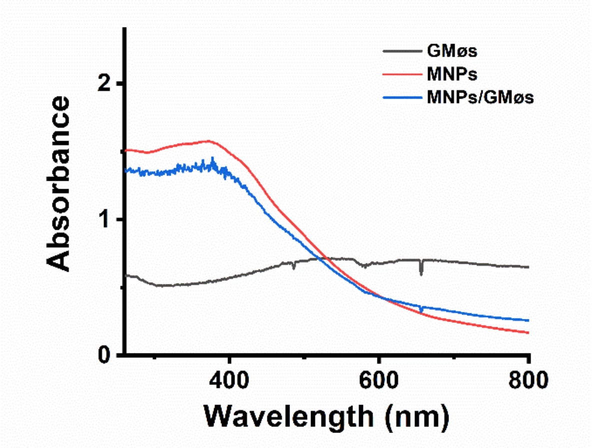

Supplementary Fig. 2: Uv/vis spectra of GMøs, MNPs, and MNPs/GMøs. The absorption characteristic peak of MNPs is located around 310-400 nm, and large cell particles don't show any characteristic peaks. The presence of MNPs peaks suggests the successful internalization of MNPs by Møs.

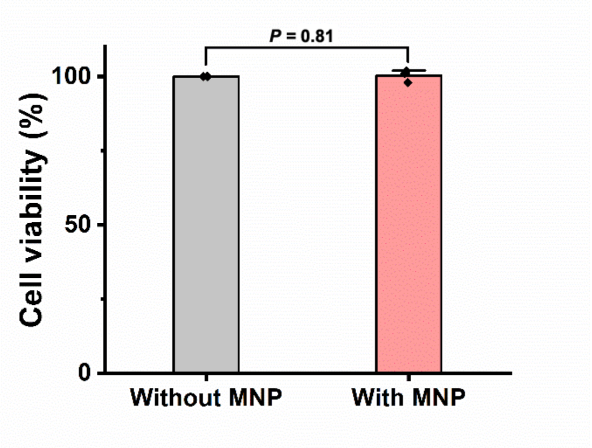

Supplementary Fig. 3: The viability of Møs without or with the treatment of MNPs (80 μg mL^{-1}) for 24 h. Statistical analysis was performed using two-sample *t* test, n.s.: not significant. The error bars represent mean ± SEM, n = 3.

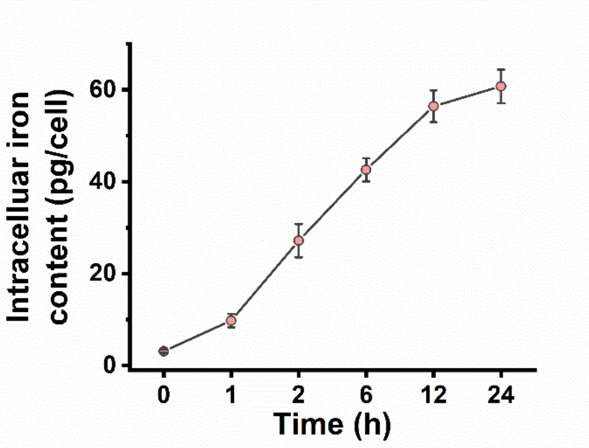

Supplementary Fig. 4: ICP-MS analysis of MNPs internalization at various times. The error bars represent mean ± SEM, n = 3.

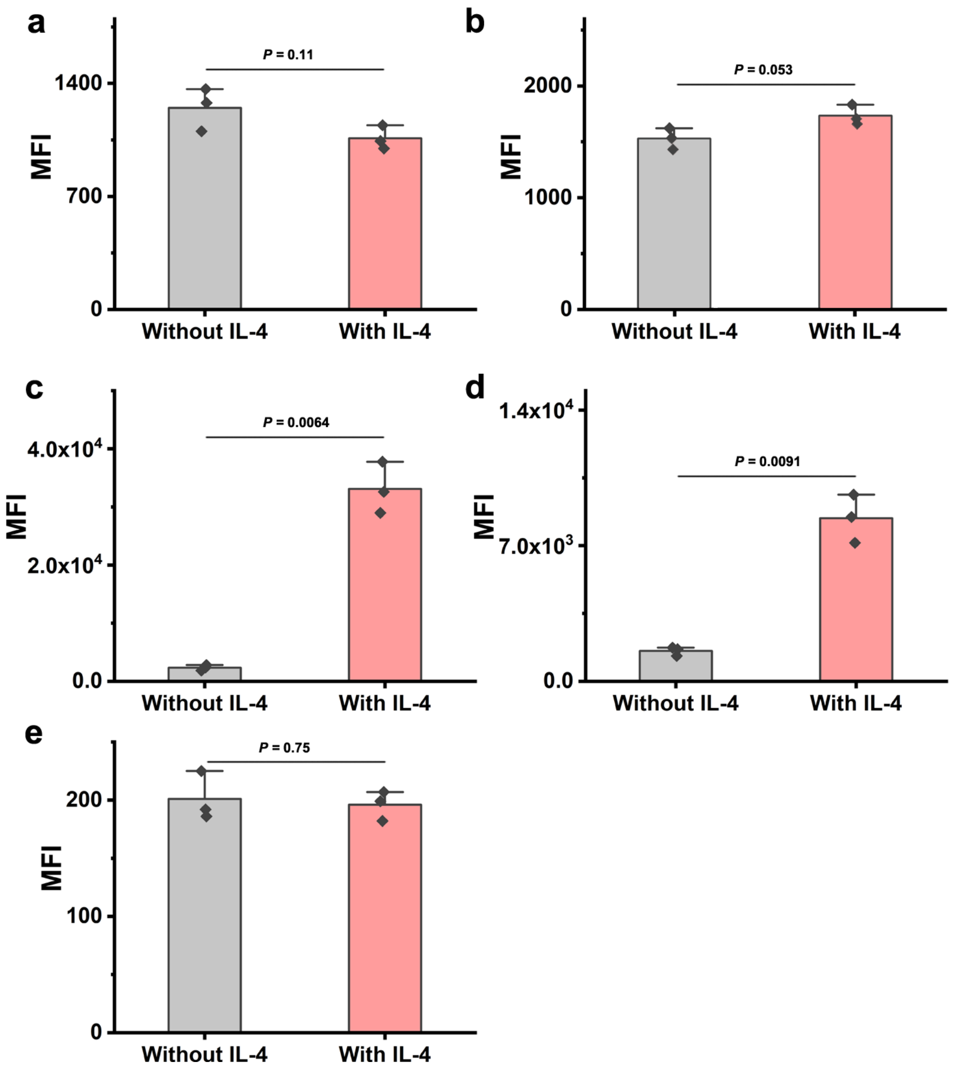

Supplementary Fig. 5: The effects of IL-4 treatment on (**a**) TLR2, (**b**) TLR4, (**c**) CD206 (MR), and (**d**) CD163 (SR) expression on the Møs membrane were detected by flow cytometry. (**e**) Cells treated with Rabbit IgG antibodies (isotype antibody) as a negative control. MFI: mean fluorescence intensity. The error bars represent mean ± SEM, n = 3. Statistical analysis was performed using two-sample *t* test.

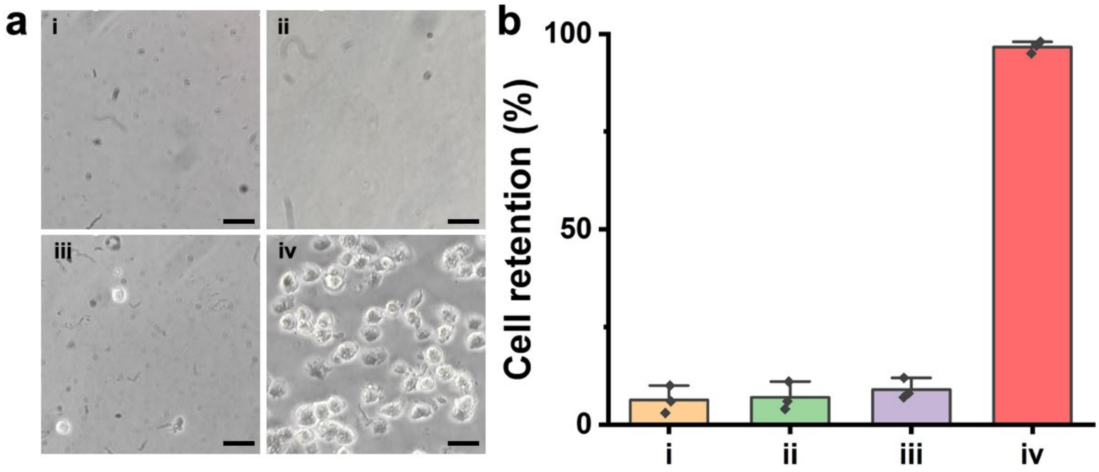

Supplementary Fig. 6: (**a**) Bright-field microscopy observations and (**b**) statistical analysis of the Møs after different treatments upon suspension in pure water. i) photoinitiator, (ii) monomer, (iii) photoinitiator + monomer, and (iv) photoinitiator + monomer + UV light. The error bars represent mean ± SEM, n = 3. Scale bars: 20 μm.

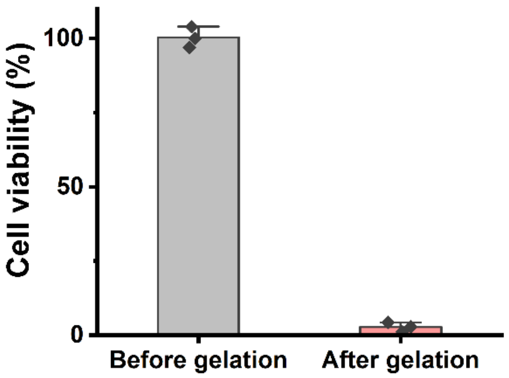

Supplementary Fig. 7: Cell viability of Møs before and after gelation. Raw 264.7 cells were cultured on a 96-well plate (1 × 10^4 cells/well). After 12 h, cells without/with gelation were treated with the 10% (v/v) CCK-8 reagent in DMEM medium for 0.5 h at 37 °C, and the absorbance at 450 nm was recorded by a microplate reader. The error bars represent mean ± SEM, n = 3.

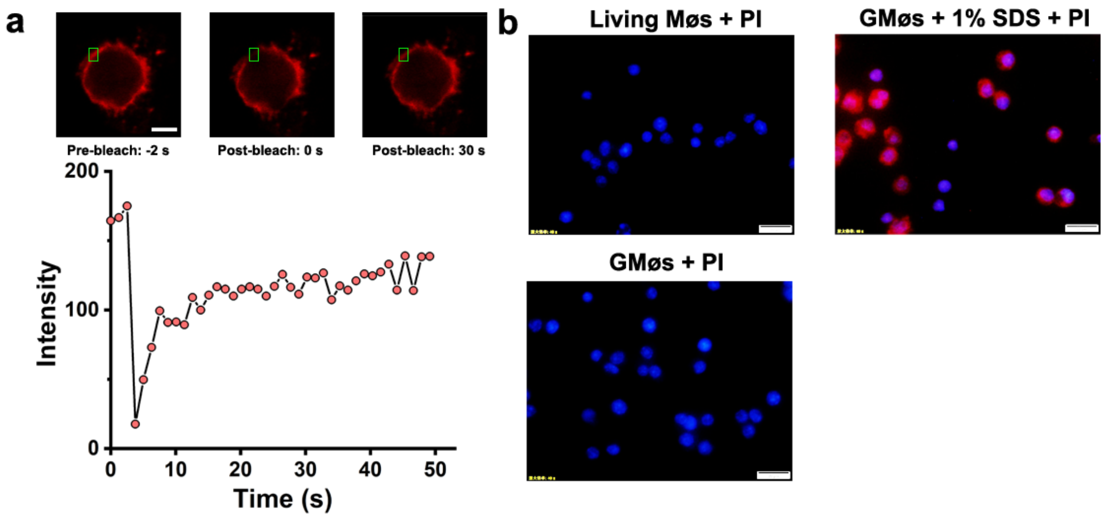

Supplementary Fig. 8: (**a**) FRAP experiment demonstrating the membrane fluidity of the prepared GMøs. Inset: Representative images showing recovery of Dil fluorescence in GMø following photobleaching. Green rectangles indicate the photobleached area of interest. Scale bars = 10 µm. (**b**) PI staining of Møs, GMøs, and GMøs pre-treated with 1% sodium dodecyl sulfate (SDS). Nucleus was stained by DAPI and shown in blue, and PI was shown in red. Scale bar: 20 µm. There is no PI fluorescence in the living Møs and GMøs, suggesting their intact cell membrane structure because of the impermeability of PI molecules. By contrast, after adding 1% SDS, the membrane of GMøs was destroyed and most cells were stained by PI molecules.

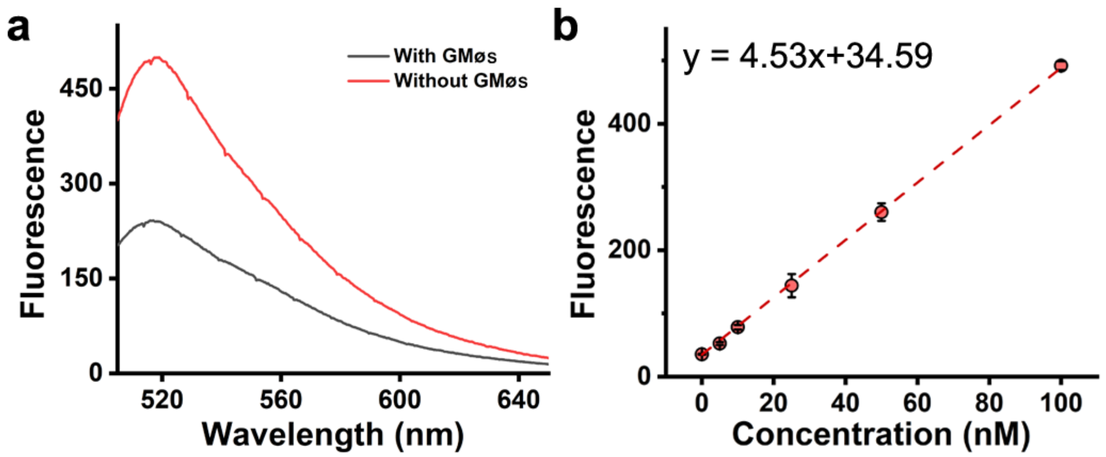

Supplementary Fig. 9: (**a**) Fluorescence spectra of Ch/FITC-DNA before and after incubating GMøs (1 × 10^7 cells). (**b**) The relationship between the concentration of Ch/FITC-DNA and fluorescence values. The error bars represent mean ± SEM, n = 3.

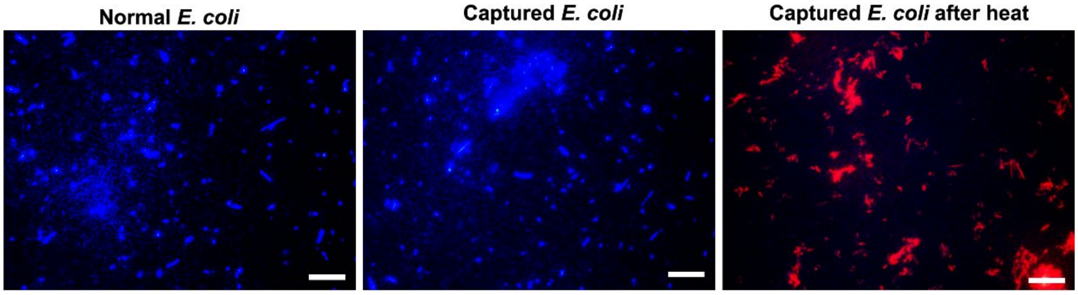

Supplementary Fig. 10: Representative fluorescence images of isolated *E. coli* by GMøs after staining with DAPI and PI dyes. DAPI fluorescence shows the location of bacteria, and PI fluorescence indicates the dead bacteria. There is no obvious difference between normal *E. coli* and captured *E. coli*, and no PI fluorescence is observed, thus verifying that the captured bacteria are still alive. Positive control shows that the captured *E. coli* killed by heat can be stained by PI. Scale bar: 20 μm.

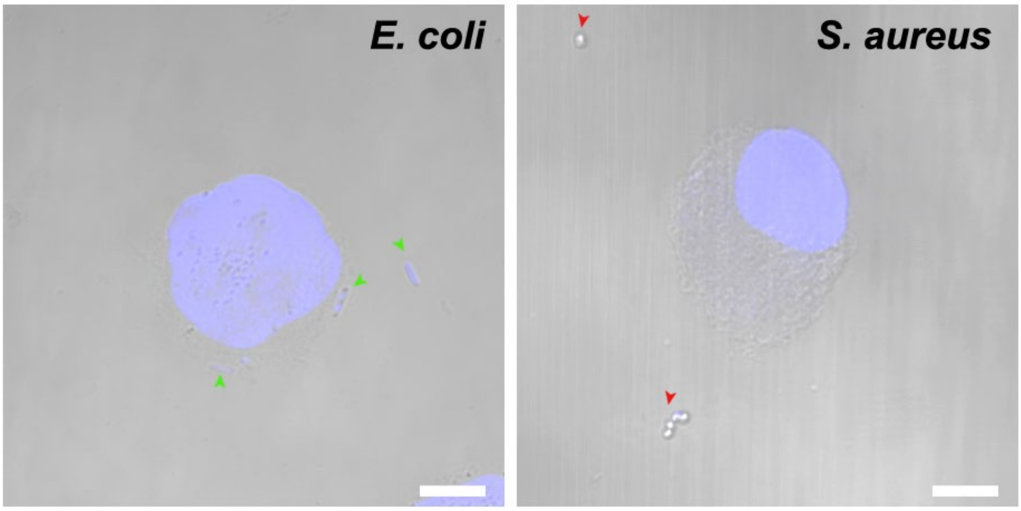

Supplementary Fig. 11: Confocal images of gelated MCF-7 cells after incubation with *E. coli* and *S. aureus*. Scale bars: 5 µm. Nuclei are shown in blue. The arrows show the location of the bacteria.

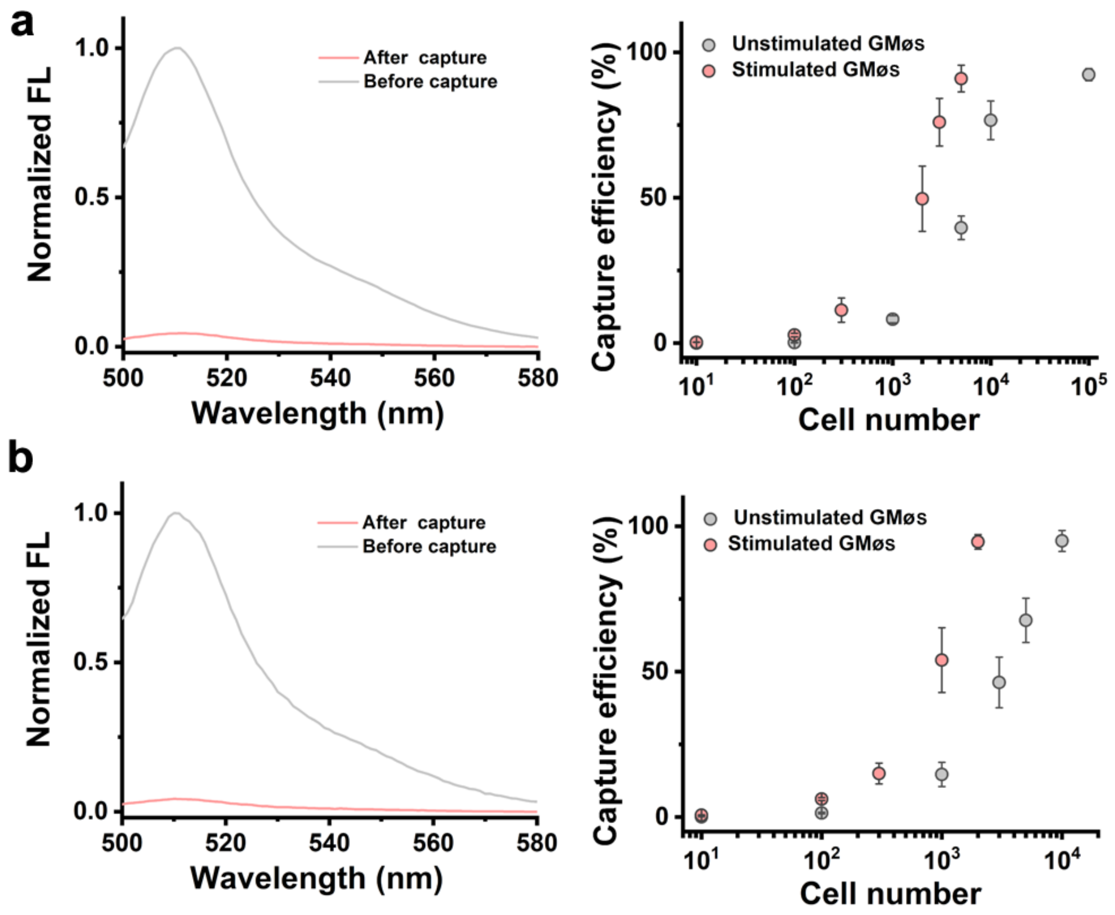

Supplementary Fig. 12: Titration assay showing the capture efficiency of unstimulated or IL-4-stimulated GMøs for (**a**) *E. coli* and (**b**) *S. aureus* capture. The number of GFP-expressing bacteria in solution is fixed to be about 10^5 cells. Fluorescence spectra showing the fluorescence change of bacteria solution before and after incubating with stimulated GMøs (10^4 GMøs). The error bars represent mean ± SEM, n = 3.

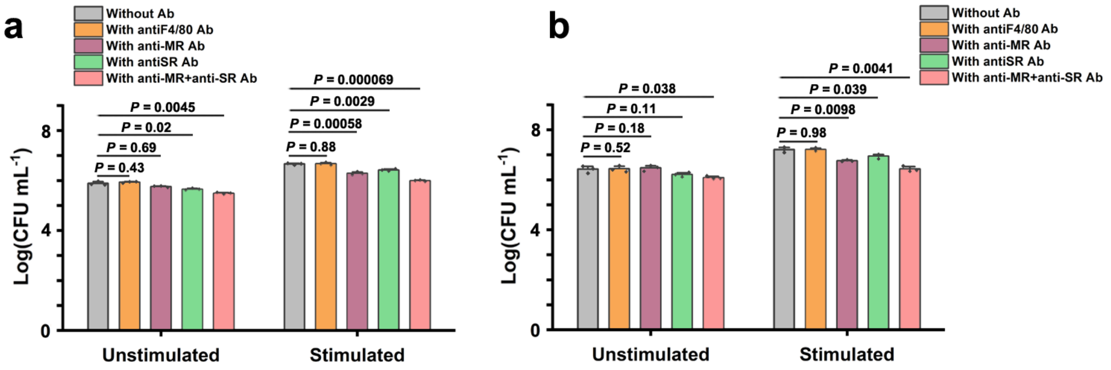

Supplementary Fig. 13: Capture of (**a**) *E. coli* and (**b**) *S. aureus* using IL-4-unstimulated/stimulated GMøs with or without pre-blockage of different antibodies. Among them, anti-F4/80 antibody is used as a negative control since F4/80 is not directly involved in bacterial capture. *S. aureus* was pre-treated with Fc fragments to avoid the interference of protein A on the bacterial surface. The error bars represent mean ± SEM, n = 3. Statistical analysis was performed using one-way analysis of variance (ANOVA).

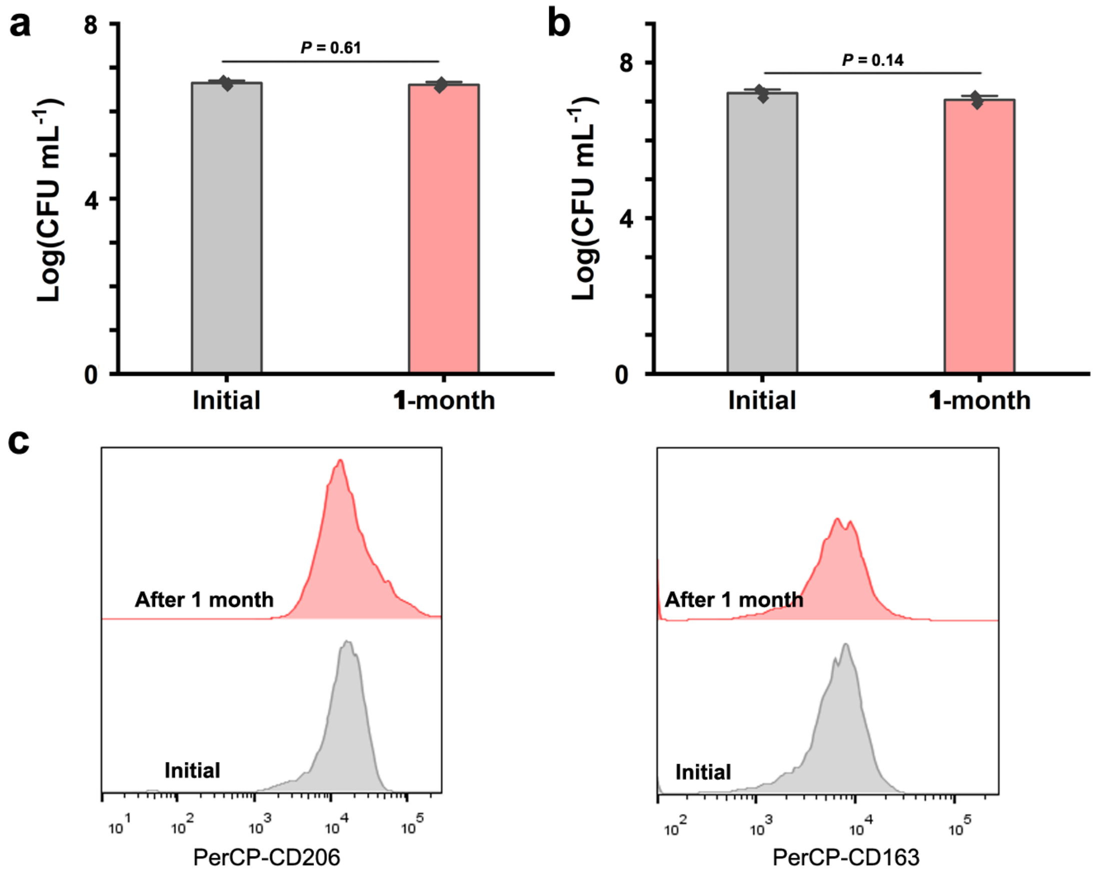

Supplementary Fig. 14: Comparison of GMøs for (**a**) *E. coli* and (**b**) *S. aureus* capture before and after being stored for 1 month. (**c**) No obvious change was seen for CD206 (left) and CD163 (right) markers determined by flow cytometry analysis after storing the GMøs for 1 month. The error bars in (Fig. 14a,b) represent mean ± SEM, n = 3. Statistical analysis in (Fig. 14a,b) was performed using two-sided pair-sample *t* test.

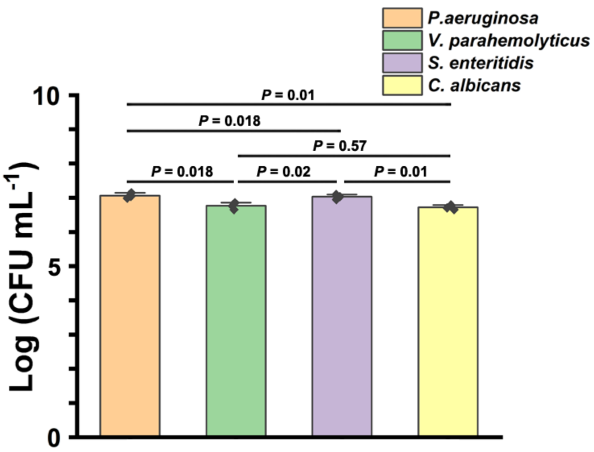

Supplementary Fig. 15: Colony statistics of different microorganisms captured by GMøs using a plate assay. The error bars represent mean ± SEM, n = 3. Statistical analysis was performed using one-way analysis of variance (ANOVA).

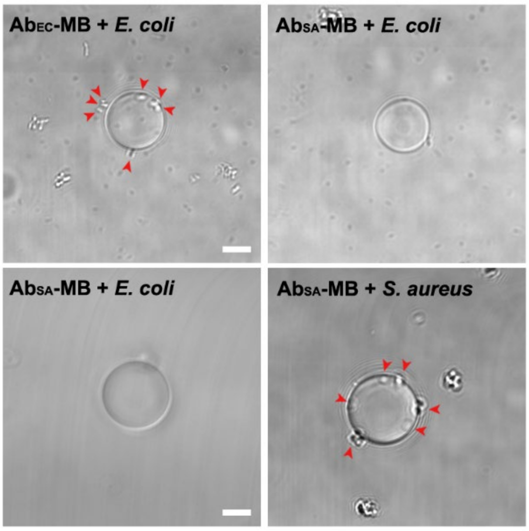

Supplementary Fig. 16: Capture of two bacteria (*E. coli* and *S. aureus*) using two antibody-modified magnetic beads (Ab-MBs, diameter = 10 µm).

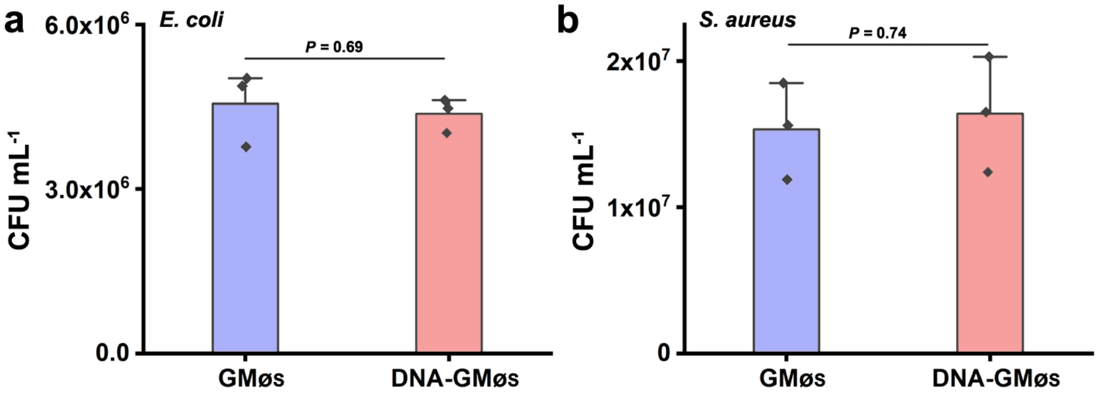

Supplementary Fig. 17: Comparison of GMøs and DNA-GMøs for capturing (**a**) *E. coli* and (**b**) *S. aureus*. The error bars represent mean ± SEM, n = 3. Statistical analysis was in (Fig. 17a,b) performed using two-sided two-sample *t* test.

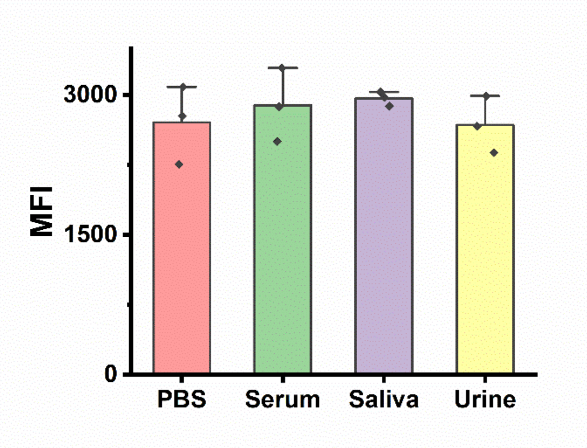

Supplementary Fig. 18: Flow cytometry results of Dz^{EC}-GMø-based assay for the detection of *E. coli* (10^6 CFU mL^{-1}) spiked in different biological media. The error bars represent mean ± SEM, n = 3. MFI: mean fluorescence intensity.

Supplementary Table 2 Comparison of the proposed method with previously reported DNAzyme-based strategies for *E. coli* detection.

**Method**	**Sensitivity (CFU mL^{-1})**	**Bacteria culture**	**Assay time (h)**	**Ref.**
Fluorescent	10^5	No	0.5	1
Fluorescent	1	Yes	12	2
Fluorescent	10^4	No	4	3
Colorimetric	10^3	No	35	4
Colorimetric	20	No	2.5	5
Colorimetric	1	Yes	7 h	6
Electrochemical	10^3	No	1	7
FC[a] or FM[b]	500 (FM analysis)<br>1 (FM analysis)	No	0.5	This work

[a]Flow cytometry analysis

[b]Flourescence microscopy analysis

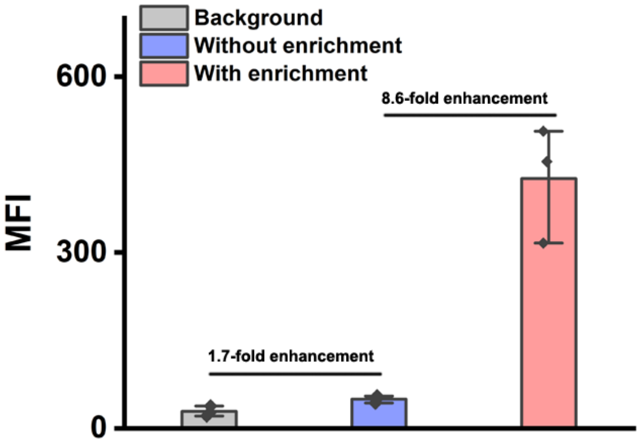

Supplementary Fig. 19: Positive role of magnetic enrichment in *E. coli* detection. A low-concentration *E. coli* (10^4 CFU mL^{-1}) was spiked in the 0.1% blood samples. After mixing the Dz^{EC}-GMøs (10^5 particles) with bacteria-containing blood samples, magnetic enrichment process was performed and the cell particles were transferred into a new tube and incubated for 30 min. Then, the cell particles were analyzed by flow cytometry analysis. As a comparison, no enrichment process was performed and the samples were directly measured by flow cytometry. The error bars represent mean ± SEM, n = 3.

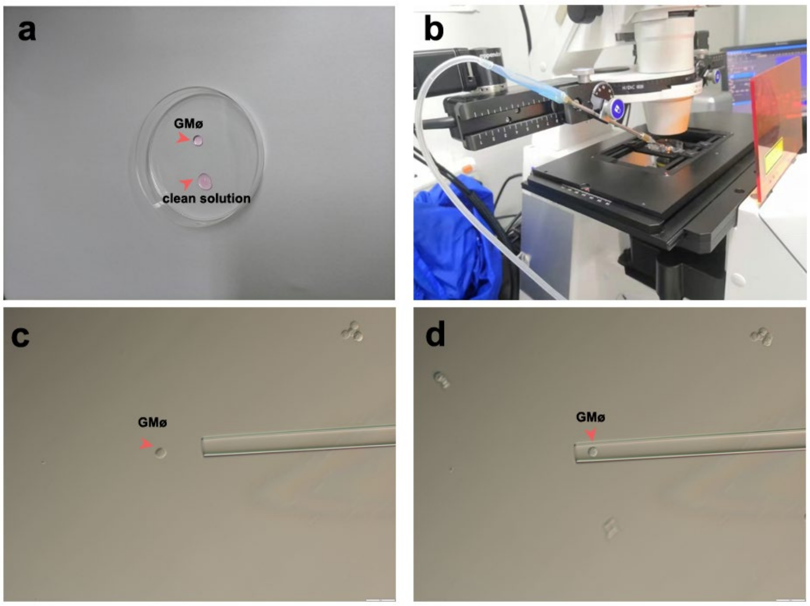

Supplementary Fig. 20: The catching process of a single-cell based reaction system. Briefly, 50 µL of solution containing GMø was kept on a transparent plate cover (**a**). Then, a Narishige micromanipulator system with a monitor is equipped on the microscope for capturing a single cell particle (**b**). As a result, one can search for and capture a suitable microbead within 2 min (**c, d**). Then, the captured particle was transferred and released into a clean drop on the plate for a single-cell particle assay.

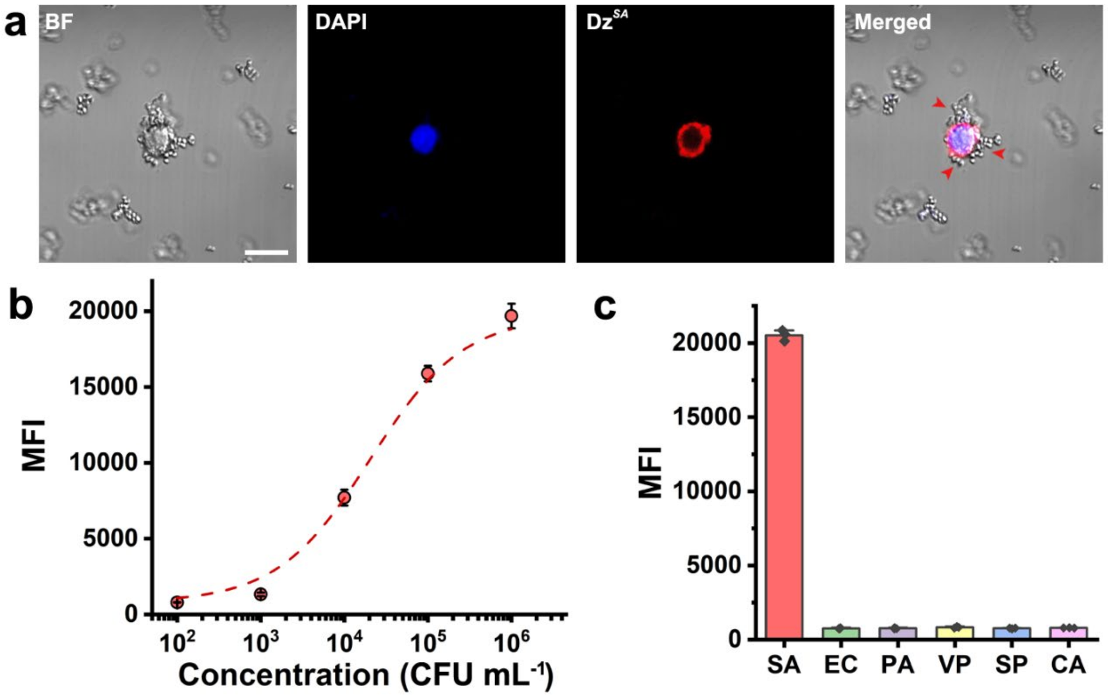

Supplementary Fig. 21: (**a**) Confocal images of a single Dz^{SA}-GMø in the presence of *S. aureus*. Scale bars: 10 µm. (**b**) Fluorescence intensity of different concentrations of *S. aureus* (0 to 10^6 CFU/mL) using a Dz^{SA}-GMø-based assay. MFI: mean fluorescence intensity. (**c**) Specificity study of Dz^{SA}-GMøs for *S. aureus* detection. *E. coli*: EC, *P. aeruginosa*: PA, *V. parahemolyticus*: VP, *S. pyogenes*: SP, and *C. albicans*: CA. The error bars represent mean ± SEM, n = 3.

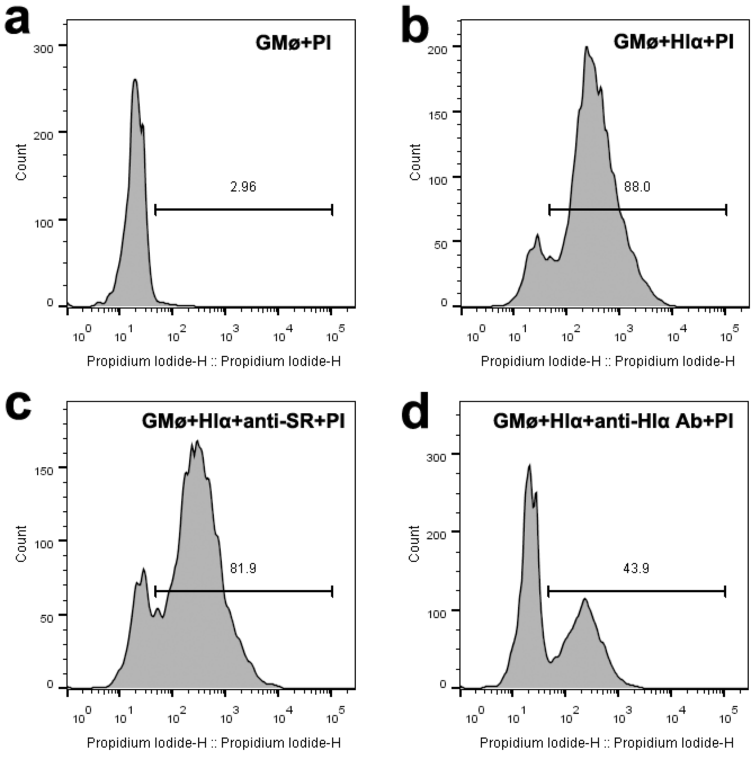

Supplementary Fig. 22: Flow cytometry analysis of GMøs with different treatments and subsequently stained by PI molecules. (**b**) GMøs treated with Hlα, (**c**) Hlα+anti-SR antibody, and (**d**) Hlα+anti-Hlα antibody for 0.5 h. (**a**) GMøs incubated with PI was used as negative control. [Hlα] = 10 nM, [antibody] = 3.3 μg mL^{-1}.

Supplementary Table 3 Comparison of the proposed method with previously reported strategies for pore-forming toxin detection.

Method	Sensitivity (nM)	Assay time (min)	Ref.
Hemolytic assay	30	75	8
MALDI-TOF MS	1	15	9
Fluorescence	50	20	10
Colorimetric	5.8	45	11
Colorimetric	0.1	30	12
Electrochemistry	0.5	1	13
SCMP	10^{-6}	30	This work

[a]Single-cell-based microscopy platform

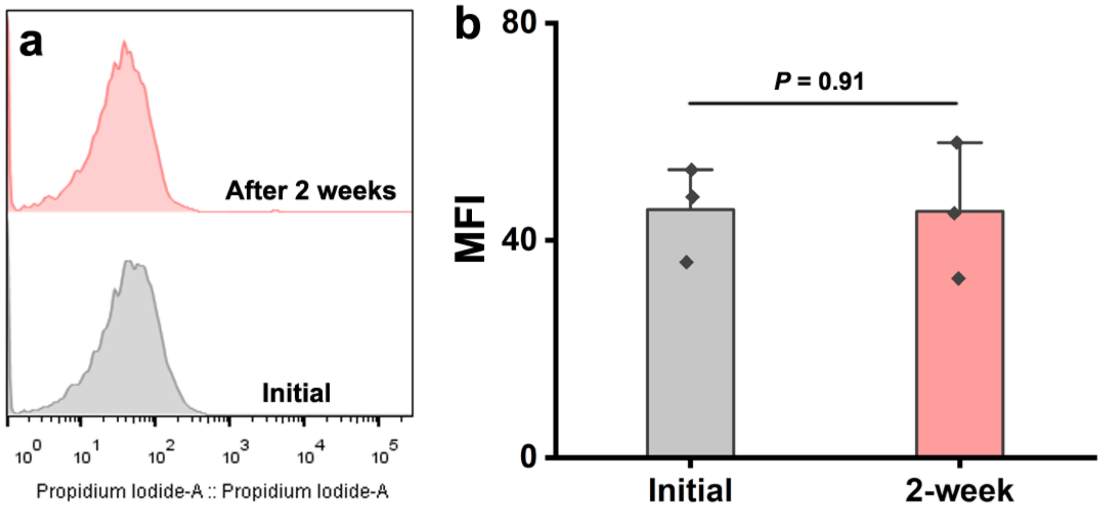

Supplementary Fig. 23: (**a**) Flow cytometry analysis and (**b**) mean fluorescence intensity of the GMøs stained by PI dyes before and after being stored for 2 weeks. The error bars represent mean ± SEM, n = 3. Statistical analysis was performed using two-sided pair-sample *t* test.

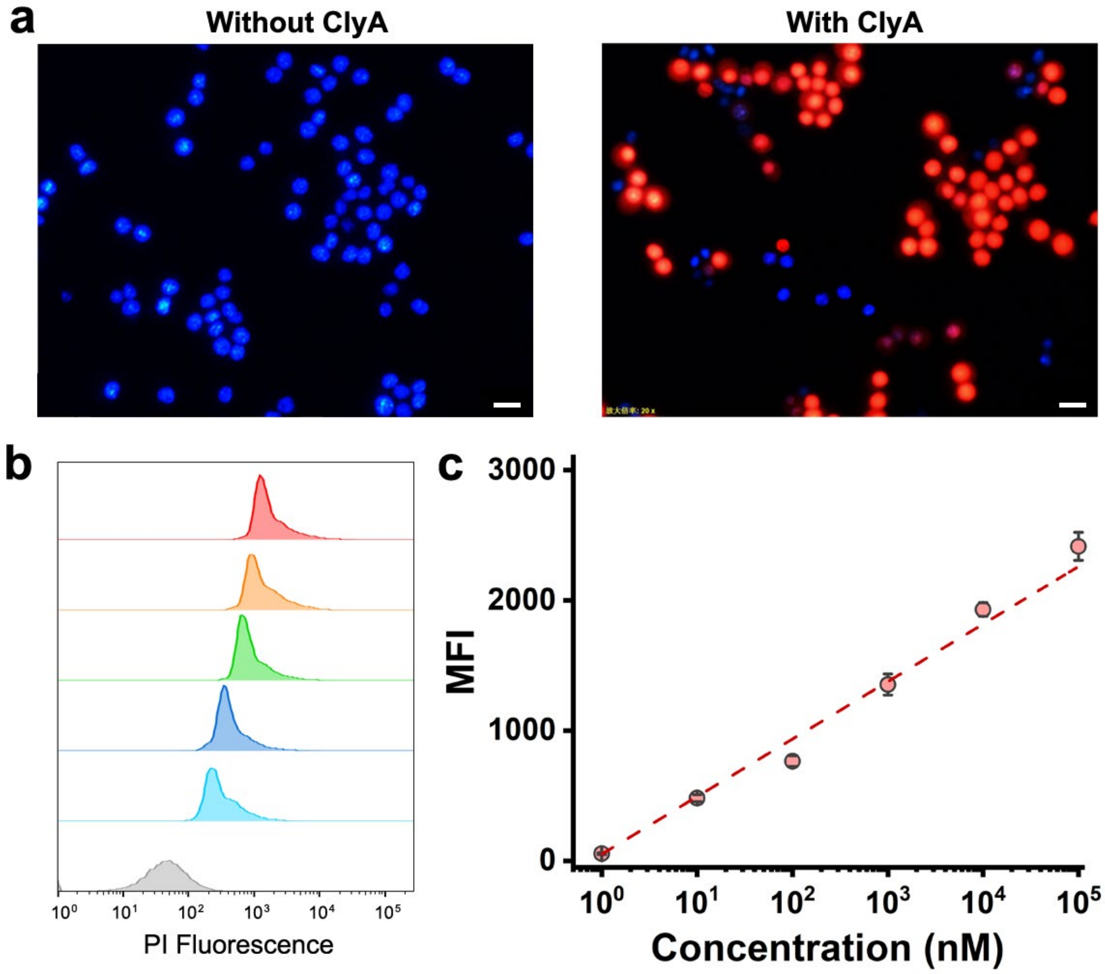

Supplementary Fig. 24: GMø-based assay for analysis of cytolysin A (ClyA) using (**a**) Fluorescence microscopy images of the PI-stained GMø particles with or without ClyA treatment (1 µM). Scale bar: 20 µm. (**b**) Flow cytometry analysis of the PI-stained GMø particles incubated with different concentrations of ClyA. From bottom to up: 0, 10, 100, 10^3, 10^4, and 10^5 nM. (**c**) Fluorescence intensities of the PI-stained GMøs incubated with different concentrations of ClyA. The error bars represent mean ± SEM, n = 3. MFI: mean fluorescence intensity.

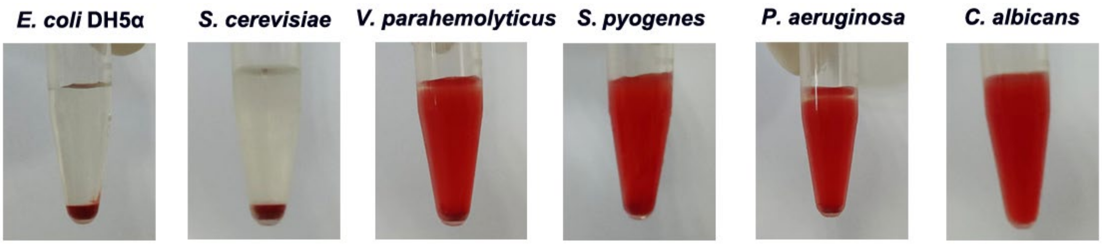

Supplementary Fig. 25: Hemolytic assay showing the disruption effect of different bacteria on red blood cells.

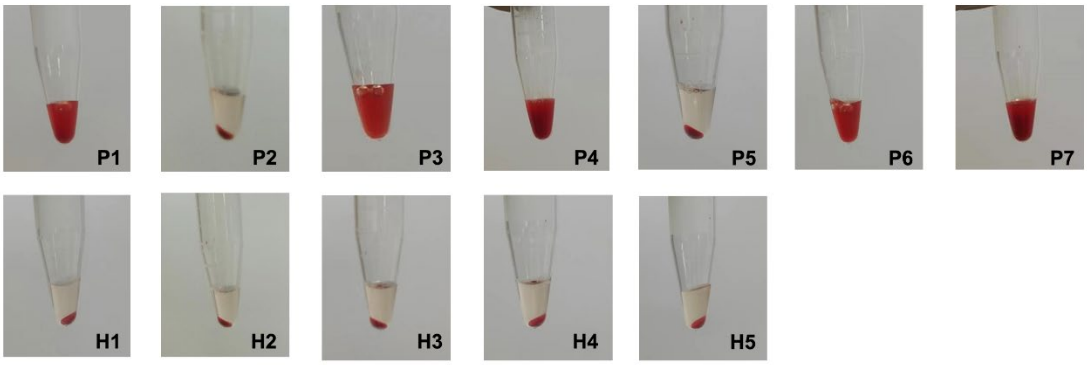

Supplementary Fig. 26: Hemolytic assay confirming the secretion of PTFs by *S. aureus* in the patients' sputum samples. Patient samples (P1-P7), healthy individuals (H1-H5).

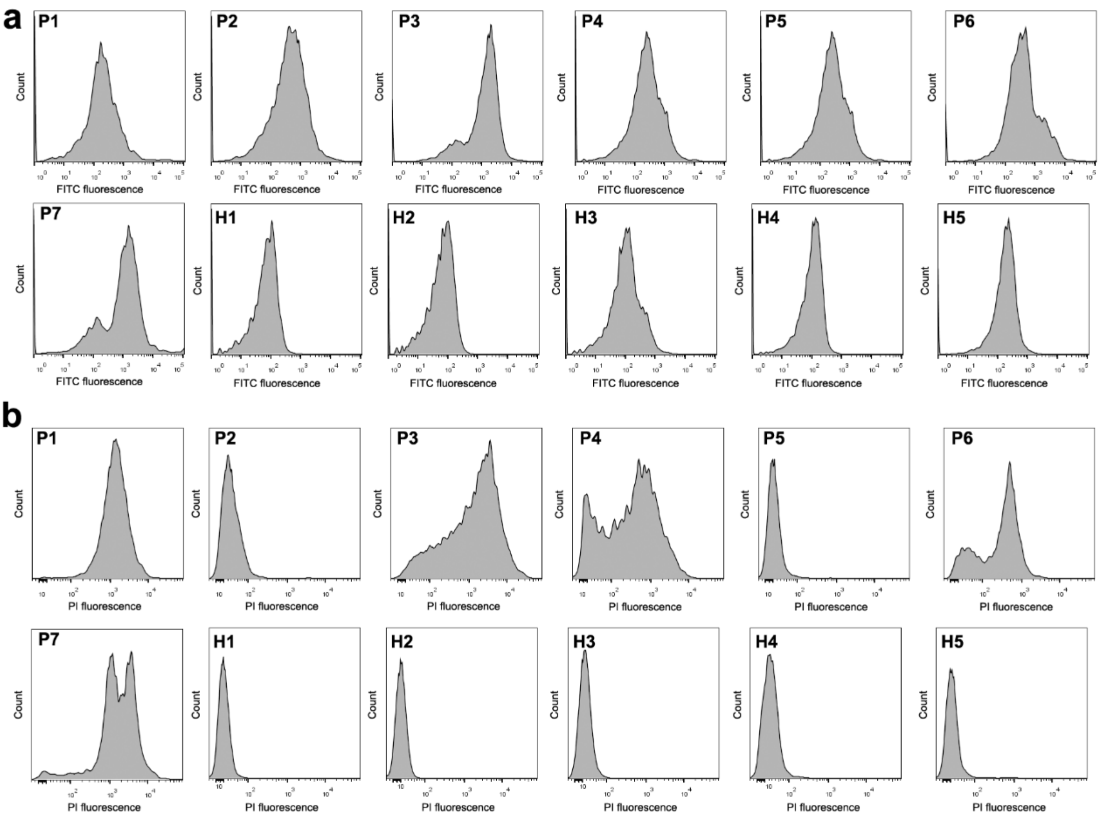

Supplementary Fig. 27: Flow cytometry analysis of (**a**) *S. aureus* (FITC fluorescence) and (**b**) Hlα (PI fluorescence) in the sputum samples using Dz^{SA}-GMø-based assay. Patient samples (P1-P7), healthy individuals (H1-H5).

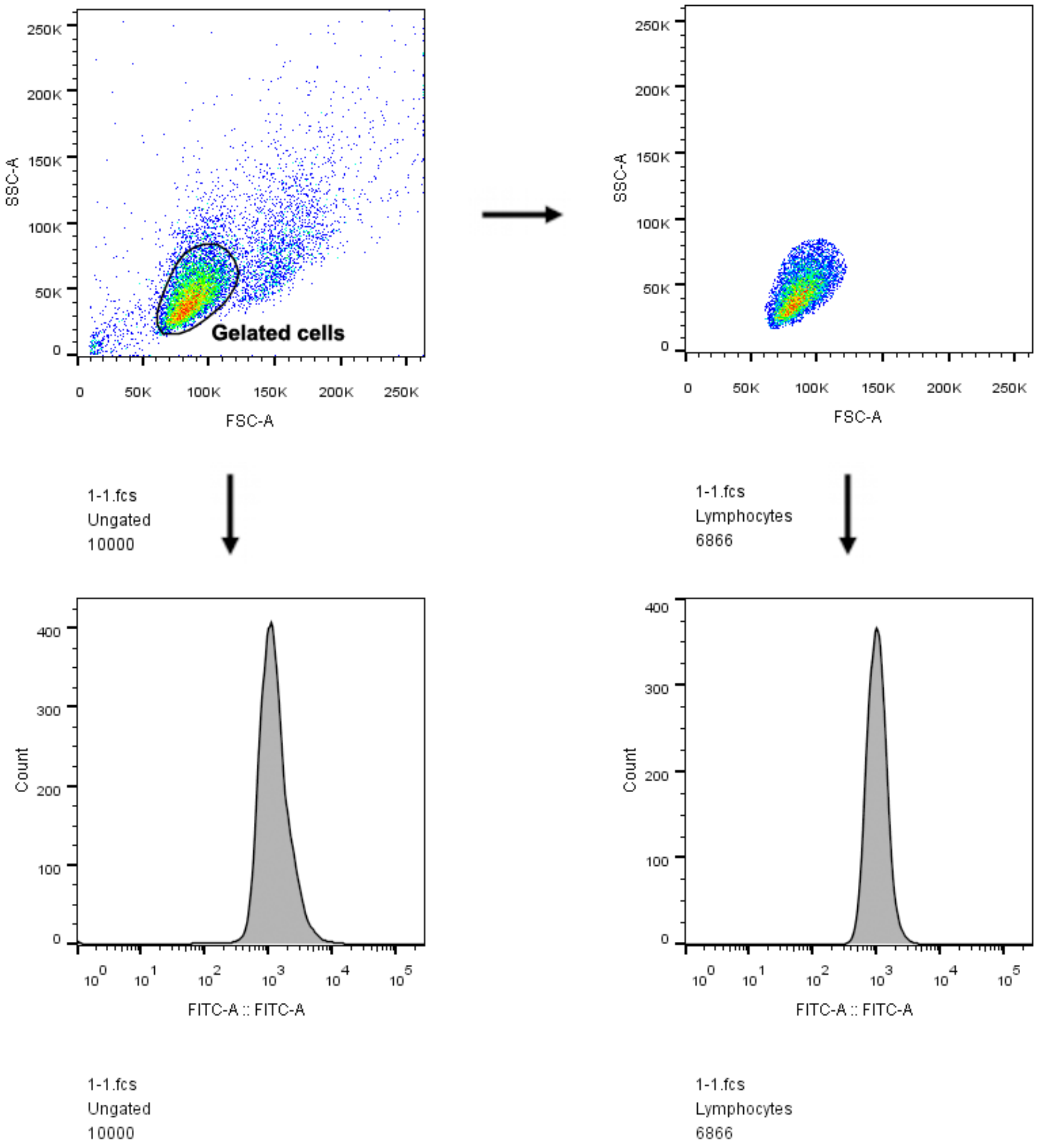

Supplementary Fig. 28: Exemplification of the gating strategy for flow cytometry analysis.

Supplementary References

1. Liu, M., Zhang, Q., Brennan, J. D. & Li, Y. F. Graphene-DNAzyme-based fluorescent biosensor for Escherichia coli detection. *MRS Commun.* **8**, 687-694 (2018).
2. Ali, M. M., Aguirre, S.D., Lazim, H. & Li, Y. F. Fluorogenic DNAzyme probes as bacterial indicators. *Angew. Chem., Int. Ed.* **50**, 3751-3754 (2011).
3. Yousefi, H., Ali, M. M., Su, H. M., Filipe, C. D. M. & Didar, T. F. Sentinel wraps: real-time monitoring of food contamination by printing DNAzyme probes on food packaging. *ACS Nano* **12**, 3287-3294 (2018).
4. Sun, Y., Chang, Y., Zhang, Q. & Liu, M. An origami paper-based device printed with DNAzyme-containing DNA superstructures for Escherichia coli detection. *Micromachines (Basel)* **10**, 531 (2019).
5. Yu, F., Li, Y., Li, M., Tang, L. & He, J. J. DNAzyme-integrated plasmonic nanosensor for bacterial sample-to-answer detection. *Biosens Bioelectron* **89**, 880-885 (2017).
6. Tram, K., Kanda, P., Salena, B. J., Huan, S. Y. & Li, Y. F. Translating bacterial detection by DNAzymes into a litmus test. *Angew. Chem., Int. Ed.* **53**, 12799-12802 (2014).
7. Pandey, R., Chang, D., Smieja, M., Hoare, T., Li, Y. & Soleymani, L. Integrating programmable DNAzymes with electrical readout for rapid and culture-free bacterial detection using a handheld platform. *Nat Chem* **13**, 895-901 (2021).
8. Soromou, L. et al. Subinhibitory concentrations of pinocembrin exert anti-S taphylococcus aureus activity by reducing α-toxin expression. *Journal of Applied Microbiology* **115**, 41-49 (2013).
9. Tonacini, J. et al. Intact Staphylococcus enterotoxin SEB from culture supernatant detected by MALDI-TOF mass spectrometry. *Toxins (Basel)* **11**, 101 (2019).
10. Li, M. L. et al. One-step assay of pore-forming biotoxins based on biomimetic perovskite nanocrystals. *Sens. Actuators B Chem.* **338**, 129839 (2021).
11. Dou, L. N. et al. Biomimetic cell model for fluorometric and smartphone colorimetric dual-signal readout detection of bacterial toxin. *Sens. Actuators B Chem.* **312**, 127956 (2020).
12. Ma, G. & Cheng, Q. Vesicular polydiacetylene sensor for colorimetric signaling of bacterial pore-forming toxin. *Langmuir* **21**, 6123-6126 (2005).
13. Valincius, G., Budvytyte, R., Penkauskas, T., Pleckaityte, M. & Zvirbliene, A. Phospholipid sensors for detection of bacterial pore-forming toxins. *ECS Transactions* **64**, 117 (2014).
