# Supplementary material for: A smart pathogen detector engineered from intracellular hydrogelation of DNA-decorated macrophages
Source: Nat Commun. 2023 May 22;14:2927. doi: 10.1038/s41467-023-38733-w (PMC10203291; doi:10.1038/s41467-023-38733-w)
Supplement: Supplementary file 3 — Reporting Summary [file 41467_2023_38733_MOESM3_ESM.pdf]

nature portfolio

Corresponding author(s): Chao Li

Last updated by author(s): Apr 25, 2023

Reporting Summary

Nature Portfolio wishes to improve the reproducibility of the work that we publish. This form provides structure for consistency and transparency in reporting. For further information on Nature Portfolio policies, see our Editorial Policies and the Editorial Policy Checklist.

Statistics

For all statistical analyses, confirm that the following items are present in the figure legend, table legend, main text, or Methods section.

n/a	Confirmed	
☐	☒	The exact sample size (*n*) for each experimental group/condition, given as a discrete number and unit of measurement
☐	☒	A statement on whether measurements were taken from distinct samples or whether the same sample was measured repeatedly
☐	☒	The statistical test(s) used AND whether they are one- or two-sided<br>*Only common tests should be described solely by name; describe more complex techniques in the Methods section.*
☒	☐	A description of all covariates tested
☒	☐	A description of any assumptions or corrections, such as tests of normality and adjustment for multiple comparisons
☐	☒	A full description of the statistical parameters including central tendency (e.g. means) or other basic estimates (e.g. regression coefficient) AND variation (e.g. standard deviation) or associated estimates of uncertainty (e.g. confidence intervals)
☐	☒	For null hypothesis testing, the test statistic (e.g. *F*, *t*, *r*) with confidence intervals, effect sizes, degrees of freedom and *P* value noted<br>*Give P values as exact values whenever suitable.*
☒	☐	For Bayesian analysis, information on the choice of priors and Markov chain Monte Carlo settings
☒	☐	For hierarchical and complex designs, identification of the appropriate level for tests and full reporting of outcomes
☒	☐	Estimates of effect sizes (e.g. Cohen's *d*, Pearson's *r*), indicating how they were calculated

Our web collection on statistics for biologists contains articles on many of the points above.

Software and code

Policy information about availability of computer code

Data collection	F-7100 fluorophotometer (Hitachi, Japan)<br>JEM-1400Flash microscope (JEOL, Japan)<br>Flow cytometry analysis (BD FACS Verse, USA)<br>Confocal microscopy analysis (Carl Zeiss LSM880, Germany)<br>Fluorescence microscopy analysis (Carl Zeiss Axio A1, Germany)<br>Microplate reader (TECAN Infinite M200, Switzerland)
Data analysis	OriginPro 2019 was used for statistical analysis, and cell images were analyzed by Image J v1.52. Confocal images were analyzed by ZEN v2.3 SP1 (blue edition). Flow cytometry data was analyzed by FACSDiva v9.0 software and Flowjo v10.0.

For manuscripts utilizing custom algorithms or software that are central to the research but not yet described in published literature, software must be made available to editors and reviewers. We strongly encourage code deposition in a community repository (e.g. GitHub). See the Nature Portfolio guidelines for submitting code & software for further information.

Data

Policy information about availability of data

All manuscripts must include a data availability statement. This statement should provide the following information, where applicable:

- Accession codes, unique identifiers, or web links for publicly available datasets
- A description of any restrictions on data availability
- For clinical datasets or third party data, please ensure that the statement adheres to our policy

The data generated in this study are provided in the Supplementary Information/ Source Data file. Data is also available from the corresponding author upon request. All the raw data used to retrieve average values are provided in an Excel file as a Source Data File.

Human research participants

Policy information about studies involving human research participants and Sex and Gender in Research.

Reporting on sex and gender	The results refer to sex (female, n= 6, male, n=6), not gender.
Population characteristics	Randomization was not relevant. Results are categorized according to diagnosis, which represents the most important covariate.
Recruitment	Eligible patients and healthy subjects (> 18 years of age) were invited to participate in this study. The inclusion criteria were diagnosed with S. aureus-caused pneumonia and received no antibiotic therapy prior to sample collection. Sputum samples were collected in a uniform manner consents from the First Affiliated Hospital of Nanjing Medical University. The presence of S. aureus was confirmed by conventional plate culture assay, and S. aureus was not detected in the sputum samples from healthy volunteers. All participates involved voluntarily and there is no participant compensation. After clear statement of the goal of the present study and how it is manipulated, we got signed consents from all participants.
Ethics oversight	Scientific Ethical Committee of the First Affiliated Hospital of Nanjing Medical University (No. 2021-SPFA-360)

Note that full information on the approval of the study protocol must also be provided in the manuscript.

Field-specific reporting

Please select the one below that is the best fit for your research. If you are not sure, read the appropriate sections before making your selection.

☒ Life sciences ☐ Behavioural & social sciences ☐ Ecological, evolutionary & environmental sciences

For a reference copy of the document with all sections, see nature.com/documents/nr-reporting-summary-flat.pdf

Life sciences study design

All studies must disclose on these points even when the disclosure is negative.

Sample size	The sample size has been indicated inthe manuscript and was determined based on prior experience and previously published works (Diaz, F. Principles and methods of validation of diagnostic assay for infectious diseases. Biology, 2009). At least three independent replicates were done in our experiments.
Data exclusions	No data were excluded.
Replication	All experiments described in this manuscript were repeated at least three times, and experimental findings were reproducible.
Randomization	All samples were randomly allocated into experimental groups.
Blinding	The investigators were not blinded to group allocation during data collection and/or analysis, because this study was observational- no intervention and exploratory in character. Blinding was not applicable to the in vitro experiments, since the investigators needed to design corresponding DNA sensing elements to detect specific bacteria. Unbiased experimental procedure and data collection/analysis were carried out as far as possible.

Reporting for specific materials, systems and methods

We require information from authors about some types of materials, experimental systems and methods used in many studies. Here, indicate whether each material, system or method listed is relevant to your study. If you are not sure if a list item applies to your research, read the appropriate section before selecting a response.

Materials & experimental systems

n/a	Involved in the study
☐	☒ Antibodies
☐	☒ Eukaryotic cell lines
☒	☐ Palaeontology and archaeology
☒	☐ Animals and other organisms
☒	☐ Clinical data
☒	☐ Dual use research of concern

Methods

n/a	Involved in the study
☒	☐ ChIP-seq
☐	☒ Flow cytometry
☒	☐ MRI-based neuroimaging

Antibodies

Antibodies used

Antibodies uesd for protein expression analysis:
1) CD206 Polyclonal antibody: 18704-1-AP, Proteintech Group, Rosemont, USA
2) CD163 Polyclonal antibody: 16646-1-AP, Proteintech Group, Rosemont, USA
3) TLR2 Polyclonal antibody: orb191498, Biorbyt, Cambridge, UK
4) TLR4 Polyclonal antibody: orb371961, Biorbyt, Cambridge, UK

Antibodies uesd for immunofluorescence imaging:
Alexa Fluor® 594-labeled CD206 monoclonal antibody (clone number: 15-2): sc-58986, Santa Cruz Biotechnology, Dallas, U.S.A.

Antibodies used for receptor blockage:
1) CD206 Polyclonal antibody: 18704-1-AP, Proteintech Group, Rosemont, USA
2) CD163 Polyclonal antibody: 16646-1-AP, Proteintech Group, Rosemont, USA
3) Alpha Hemolysin (hly) Polyclonal Antibody:abx109435, Abbexa, Cambridge, UK
4) F4/80 Polyclonal antibody, 29414-1-AP, Proteintech Group, Rosemont, USA
5) Rabbit IgG control Polyclonal antibody: 30000-0-AP, Proteintech Group, Rosemont, USA

Antibodies used for bacteria capture:
1) Anti-E. coli monoclonal antibody (clone number: 1011): sc-57709, Santa Cruz Biotechnology, Dallas, U.S.A.
2) Anti-MRSA monoclonal antibody (clone number: NYR MRSA16): sc-73327, Santa Cruz Biotechnology, Dallas, U.S.A.

Validation

All antibodies were validated by the suppliers.
CD206 Polyclonal antibody: 18704-1-AP
https://www.ptgcn.com/Products/MRC1-Antibody-18704-1-AP.htm

CD163 Polyclonal antibody: 16646-1-AP
https://www.ptgcn.com/products/CD163-Antibody-16646-1-AP.htm

TLR2 Polyclonal antibody: orb191498,
https://biorbyt.com.cn/tlr2-antibody-orb191498.html

TLR4 Polyclonal antibody: orb371961
https://biorbyt.com.cn/tlr4-antibody-orb371961.html

Alpha Hemolysin (hly) Antibody:abx109435
https://www.abbexa.com/alpha-hemolysin-antibody

F4/80 Polyclonal antibody, 29414-1-AP
https://www.ptgcn.com/products/F4-80-Antibody-29414-1-AP.htm

Goat Anti-Rabbit IgG: CW0103
https://www.cwbio.com/goods/index/id/10119

Rabbit IgG control Polyclonal antibody: 30000-0-AP,
https://www.ptgcn.com/products/IgG-control-Antibody-30000-0-AP.htm

Anti-E. coli antibody: sc-57709
https://www.scbt.com/p/e-coli-antibody-1011?productCanUrl=e-coli-antibody-1011

Anti-MRSA monoclonal antibody: sc-73327
https://www.scbt.com/p/mrsa-antibody-nyr-mrsa16

Eukaryotic cell lines

Policy information about cell lines and Sex and Gender in Research

Cell line source(s)

RAW264.7 cells,MCF-7 cells, and all bacteria strains were obtained from ATCC

Authentication | All cell lines were authenticated by short tandem repeat (STR) profiling analysis.

Mycoplasma contamination | All cell lines tested negative for mycoplasma contamination.

Commonly misidentified lines (See ICLAC register) | No commonly misidentified lines were used in this study.

Flow Cytometry

Plots

Confirm that:

☒ The axis labels state the marker and fluorochrome used (e.g. CD4-FITC).

☒ The axis scales are clearly visible. Include numbers along axes only for bottom left plot of group (a 'group' is an analysis of identical markers).

☒ All plots are contour plots with outliers or pseudocolor plots.

☒ A numerical value for number of cells or percentage (with statistics) is provided.

Methodology

Sample preparation

For stability study, freshly prepared DNA1/DNA2-decorated GMøs (1 × 10E6 cells) were incubated in 10% fetal bovine serum for 0, 1, 2 and 4 h. Then, the cell particles were collected and immediately subjected to flow cytometry analysis.
For bacteria detection, 90 µL of DNAzyme modified-GMøs (Dz-GMøs, 1 × 10E4 cells) in a reaction buffer (50 mM HEPES, 150 mM NaCl, 15 mM MgCl2, pH 7.4) was mixed with 10 µL of E. coli K12 or MRSA with varying concentrations. After 30 min of reaction, cell particles were collected and immediately subjected to flow cytometry analysis.

Instrument

BD FACSVerse, USA

Software

FACSDiva v9.0 software and Flowjo v10.0.

Cell population abundance

The cell population abundance could not be determined because the use instrument was an analytical flow cytometry without sorting system. During sample measurements, the initial gate was used to ensure a cell count of 10,000 cells was collected for a relevant cell population.

Gating strategy

Cells were gated by FSC/SSC gates to select gelated cells. Then, the fluorescent intensity of fluorescein isothiocyanate or rhodamine was detected.

☒ Tick this box to confirm that a figure exemplifying the gating strategy is provided in the Supplementary Information.
